# Activation of nuclear factor‐κB in the angiogenesis of glioma: Insights into the associated molecular mechanisms and targeted therapies

**DOI:** 10.1111/cpr.12929

**Published:** 2020-12-10

**Authors:** Jiajie Tu, Yilong Fang, Dafei Han, Xuewen Tan, Haifeng Jiang, Xun Gong, Xinming Wang, Wenming Hong, Wei Wei

**Affiliations:** ^1^ Institute of Clinical Pharmacology Key Laboratory of Anti‐Inflammatory and Immune Medicine Ministry of Education Anhui Collaborative Innovation Center of Anti‐Inflammatory and Immune Medicine Anhui Medical University Hefei China; ^2^ Department of Neurosurgery The First Affiliated Hospital of Anhui Medical University Hefei China

**Keywords:** angiogenesis, Glioma, NF‐κB

## Abstract

Glioma is the most commonly observed primary intracranial tumour and is associated with massive angiogenesis. Glioma neovascularization provides nutrients for the growth and metabolism of tumour tissues, promotes tumour cell division and proliferation, and provides conditions ideal for the infiltration and migration of tumour cells to distant places. Growing evidence suggests that there is a correlation between the activation of nuclear factor (NF)‐κB and the angiogenesis of glioma. In this review article, we highlighted the functions of NF‐κB in the angiogenesis of glioma, showing that NF‐κB activation plays a pivotal role in the growth and progression of glioma angiogenesis and is a rational therapeutic target for antiangiogenic strategies aimed at glioma.

## Graphical abstract

Growing evidence suggests that there is a correlation between the activation of nuclear factor (NF)‐κB and the angiogenesis of glioma. Herein, we sought to discuss the current understanding of the molecular mechanisms of NF‐κB in diverse glioma microenvironments such as hypoxia, inflammation, and oxidative stress, and its function as a therapeutic target for antiangiogenic strategies aimed at glioma.

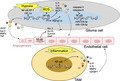

## 1 INTRODUCTION

Glioma, which originates from the glial cells surrounding the neurons, is the most commonly observed intracranial tumour with the greatest degree of malignancy and accounts for approximately 80% of all brain malignancies.^1^ The median survival of malignant glioma patients is only about 1 year, even after common treatments including surgical resection, radiotherapy and chemotherapy are performed.^2^ Angiogenesis is among the factors vital to tumour development.^3^ As is the case with most solid tumours, the survival and growth of fast‐growing gliomas with an avascular area volume exceeding 2 mm^3^ require newly generated blood vessels for the provision of the necessary oxygen, growth factors and nutrients.^4^ Several studies have shown that angiogenesis has the strongest prognostic significance among all the clinical and pathological features of glioma, and that widespread angiogenesis tends to be associated with worse prognoses.^5^ Based on the clinical significance and potentialities of the therapeutic interventions for glioma, it is necessary to identify the targets and underlying molecular mechanisms that regulate glioma angiogenesis.

Tumour necrosis factor (TNF, also referred to as TNF‐α) exerts its function using two receptors—TNF receptor I (TNFR1, p55 receptor) and TNF receptor II (TNFR2, p75 receptor)—which are members of the TNF superfamily.^6^ TNF plays a role in the promotion of tumour cell apoptosis through TNFR1 binding; however, it also promotes tumour cell growth through TNFR2.^7^, ^8^ TNFR2 activation leads to the recruitment of TNF receptor‐associated factor 2 and motivates the pro‐survival nuclear factor (NF)‐κB pathway.^9^, ^10^ NF‐κB regulates the genes involved in tumour microenvironment development and proangiogenic and pro‐inflammatory cytokine synthesis.^11^ Abnormal or constitutive NF‐κB activity in glioma^12^ and a remarkable correlation between NF‐κB activation level and glioma grade have been previously demonstrated.^13^ Furthermore, accumulating evidence shows that constitutive NF‐κB activity could regulate the proangiogenic context of glioma. Notably, NF‐κB restraint even led to the blocking of the angiogenesis of glioma in nude mice.^14^

Herein, we sought to discuss the current understanding of the molecular mechanisms of NF‐κB in diverse glioma microenvironments such as hypoxia, inflammation and oxidative stress, and its function as a therapeutic target for antiangiogenic strategies aimed at glioma.

## 2 MOLECULAR MECHANISMS AND TARGETED THERAPIES

### 2.1 NF‐κB in hypoxia‐induced glioma angiogenesis

During the entire process of angiogenesis, new capillaries sprout from the current capillaries, and endothelial cells (ECs) are released from their stroma and migrate and transfer to areas without capillaries, thereby allowing them to differentiate into tubular structures. The newly generated capillaries provide a large amount of necessary oxygen and nutrients for fast‐growing malignant tumours that have an avascular area volume greater than 2 mm.^315^

**Figure 1:**
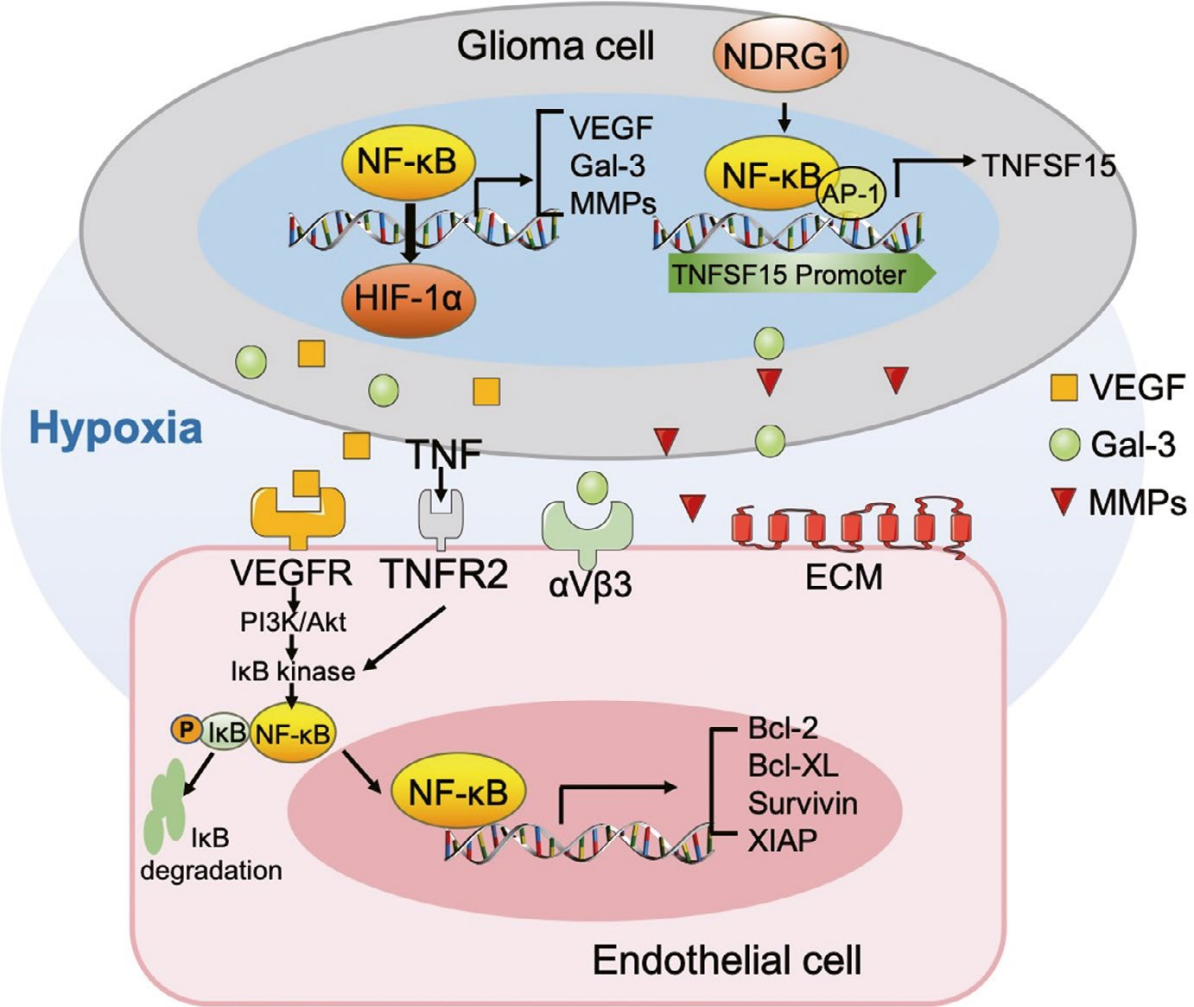
Role of NF‐κB in glioma angiogenesis under a hypoxic microenvironment. Hypoxia‐induced NF‐κB is conducive to the upregulation of the HIF‐1α, VEGF, gal‐3 and MMP genes, which are released by tumour cells to induce EC chemotaxis and motility and stimulate angiogenesis. Hypoxia activated TNFR2 and VEGFR induced NF‐κB to upregulate the expression of antiapoptotic genes, such as Bcl‐2, Bcl‐XL, survivin and XIAP in ECs. NDRG1 induced the upregulation of anti‐angiogenesis gene TNFSF15 by the activation of NF‐κB and AP‐1 in the TNFSF15 promoter region. TNFSF: tumour necrosis factor super family; TNFR: tumour necrosis factor receptor; NF‐κB: nuclear factor‐κB; VEGF: vascular endothelial growth factor; VEGFR: vascular endothelial growth factor receptor; PI3K: phosphatidylinositol 3‐kinase; Gal‐3: galectin‐3; XIAP: X‐chromosome‐linked inhibitor of apoptosis protein; HIF: hypoxia‐inducible factor; MMP: matrix metalloproteinase; AP: activator protein; ECM: extracellular matrix; NDRG1: N‐myc downstream‐regulated gene‐1

Due to abnormalities in the structure of malignant tumours, local or temporary hypoxia and a lack of nutritional components may lead to EC apoptosis and tumour angiogenesis inhibition.^16^, ^17^ Nevertheless, migrating ECs often overcome these adverse conditions to boost tumour angiogenesis. ECs are stimulated by vascular endothelial growth factor (VEGF) or adhere to extracellular matrix (ECM) molecules, leading to the augmentation of anti‐apoptotic genes via the phosphatidylinositol 3‐kinase (PI3K)/Akt or NF‐κB signalling pathways.^18^, ^19^ Akt induces the transcription function of NF‐κB by stimulating the RelA/p65 transactivation subunit via IκB kinase and activation of the protein kinase p38.^20^ Studies have reported that the induction of cell survival signals by PI3K/Akt partially mediates the activation of NF‐κB transcription factors.^20^ The VEGF released by glioma cells stimulates EC proliferation, resulting in angiogenesis.^21^ Interestingly, TNF, which could induce the apoptosis of ECs, was detected in glioma but did not inhibit the associated angiogenesis.^22^, ^23^ It was reported that human umbilical vein ECs must activate NF‐κB in order to avoid undergoing TNF‐induced apoptosis.^24^ Using a human brain microvascular endothelial cell (HBMVEC) and U251 glioma cell co‐culture system, investigators found that EC apoptosis was induced by serum starvation and reversed by recombinant VEGF protein and a culture medium of hypoxic U251 glioma cells. In addition, hypoxia treatment activated TNF‐induced VEGF and NF‐κB to upregulate the antiapoptotic gene expressions, such as those of Bcl‐2, Bcl‐XL, survivin and X‐chromosome‐linked inhibitor of apoptosis protein (XIAP) in ECs^25^ (Figure 1). Therefore, it is clear that the hypoxic environment of glioma, in addition to not killing ECs, promotes NF‐κB‐dependent angiogenesis.

Galectin‐3 (gal‐3) is a b‐galactoside binding protein that is involved in several types of pathological tumour progression, such as angiogenesis, cell proliferation and anti‐apoptosis.^26^, ^27^, ^28^ Evidence shows that gal‐3 is visibly upregulated in a hypoxia‐inducible factor (HIF)‐1α‐dependent manner in mouse fibroblasts and nucleus pulposus cells under hypoxic conditions.^29^, ^30^ In addition, HIF‐1α is a pivotal transcriptional regulator of the hypoxic response, which upregulates its target genes including vascular endothelial growth factor (VEGF) and matrix metalloproteinase (MMP) to boost tumour angiogenesis and invasion.^31^ Hypoxia is a commonly observed feature of solid tumours such as gliomas, in which a high proportion of gal‐3 accumulates.^32^, ^33^ Gal‐3 is released by tumour cells for the induction of EC chemotaxis and motility and the stimulation of angiogenesis (Figure 1). Gal‐3 knockout U87 glioma cells implanted subcutaneously in nude mice blocked tumour growth *in vivo*.^34^ In vitro, Ikemori et al found that gal‐3 protected T98G glioma cells from apoptosis in the absence of oxygen and nutrition, and the knockdown of gal‐3 induced double apoptosis. It is worth noting that the upregulation of gal‐3 was dependent not only on HIF‐1α but also on NF‐κB.^35^ Hypoxia‐induced NF‐κB was conducive to the regulation of the HIF‐1α and gal‐3 genes and prevention of cell death caused by hypoxia.^36^, ^37^ Based on the above‐stated literature, it can be concluded that hypoxia in glioma protects both ECs and tumour cells against death, which facilitates angiogenesis and leads to tumour aggravation, directly or indirectly.

N‐myc downstream‐regulated gene‐1 (NDRG1) is considered a regulatory gene in the hypoxic microenvironment of glioma.^38^ In untreated glioma patients, high NDRG1 expression was associated with increased survival, and the gene also reduced the rate of angiogenesis.^39^ Thomas et al indicated that the expression of NDRG1 was markedly upregulated during hypoxia in glioma, and that an NDRG1‐overexpressing glioma implantation model with reduced angiogenic activity reduced the rate of glioma growth and resistance to antiangiogenic treatment. The anti‐angiogenesis gene TNFSF15 showed a 30‐fold increase in glioma development, with an increasing expression of NDRG1, and demonstrated that anti‐angiogenesis was positively correlated with TNFSF15. Interestingly, mutated NF‐κB and activator protein (AP‐1) in the TNFSF15 promoter region reversed the anti‐angiogenesis of NDRG1^40^ (Figure 1). The research demonstrated that NF‐κB and AP‐1 are positively correlated with TNFSF15 expression in glioma.

Based on the aforementioned evidence, hypoxia promotes NF‐κB‐dependent angiogenesis in glioma. However, the hypoxic regulatory gene NDRG1 induces the upregulation of the anti‐angiogenesis gene TNFSF15 also depends on the transcriptional activity of NF‐κB. Therefore, NF‐κB exerts multiple effects on the angiogenic system in glioma under a hypoxic microenvironment.

### 2.2 NF‐κB in inflammation‐induced glioma angiogenesis

**Figure 2:**
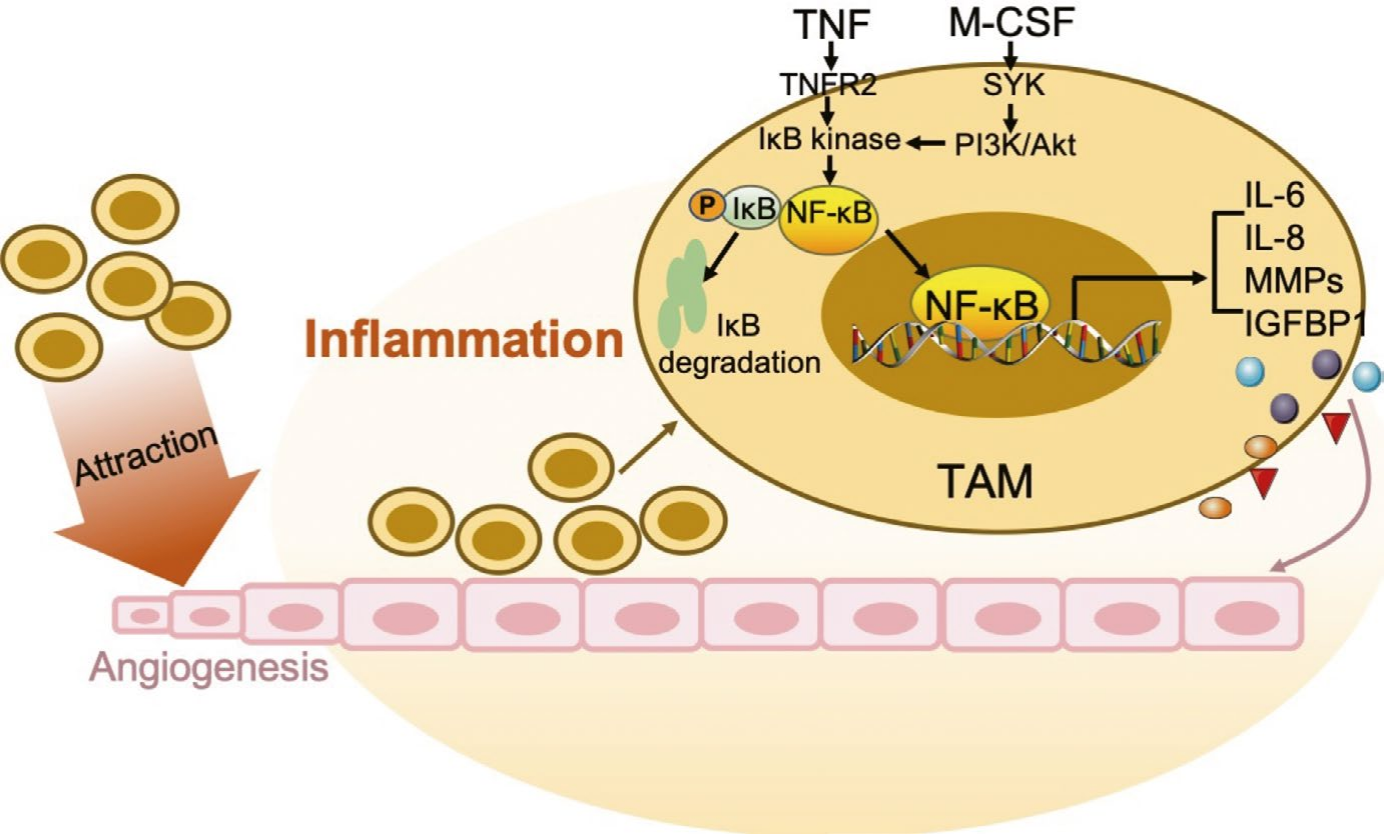
Role of NF‐κB in glioma angiogenesis under an inflammatory microenvironment. TNF activates the transcriptional activity of NF‐κB for IL‐6, IL‐8, MMPs and IGFBP1, by TNFR2, leading to angiogenesis. The newly formed blood vessels are allowed to recruit a larger number of inflammatory cells such as macrophages, leading to the further release of proangiogenic cytokines. TAM: tumour‐associated macrophages; M‐CSF: macrophage colony‐stimulating factor; SYK: spleen tyrosine kinase; IGFBP: insulin‐like growth factor binding protein; IL: interleukin; TNF: tumour necrosis factor; TNFR: tumour necrosis factor receptor; PI3K: phosphatidylinositol 3‐kinase; NF‐κB: nuclear factor‐κB; MMP: matrix metalloproteinase

Some interactions exist between inflammation and angiogenesis in the course of glioma progression.^41^ The angiogenesis of glioma depends on the interaction between tumour cells, ECs and surrounding inflammatory cells. In the tumour environment, newly formed blood vessels are allowed to recruit inflammatory cells continuously, leading to the release of proangiogenic cytokines, including VEGF‐A, MMP, chemokines and pro‐inflammatory factors. In this manner, a larger number of blood vessels are formed, ending in a vicious cycle.^42^, ^43^ The process requires the induction of a large number of pro‐inflammatory factors, such as TNF, interleukin (IL)‐8 and IL‐6, leading to capillary budding. NF‐κB, activated by TNF through the phosphorylation of IκB,^44^ binds to the promoters of IL‐8 and IL‐6 to activate them^45^, ^46^, ^47^ (Figure 2).

IL‐8 is a chemical attractant cytokine that attracts and activates neutrophils at the site of inflammation and has an angiogenic effect.^47^ It has been reported that the binding of IL‐8 to its receptors in ECs in vitro, CXCR1 and CXCR2, can activate ECs and facilitate tumour angiogenesis.^48^, ^49^ The expression level of IL‐8 is directly related to the degree of glioma angiogenesis. Furthermore, Xie et al used NF‐κB inhibitors to suppress IL‐8 secretion in vitro and *in vivo*.^14^ Studies focusing on IL‐6 in vitro indicated that it affects the human inflammatory response and is related to tumour angiogenesis through the transcriptional activity of angiogenic growth factors such as VEGF and MMP.^50^, ^51^ Meanwhile, the IL‐4 and IL‐10 released by microglia have anti‐inflammatory effects in the glioma environment.^52^ The IL‐4 released by macrophages can be used as anti‐inflammatory agents of TNF.^53^ Importantly, IL‐4 inhibits the migration of ECs to inflammatory regions and inhibits their differentiation into organized vascular structures.^54^

As mentioned above, angiogenesis is the formation of new capillaries based on existing blood vessels and is the result of inflammation. Macrophages are among the most important inflammatory immune cells in the tumour matrix. Tumour‐associated macrophages (TAMs) are recruited to the periphery of tumour cells, have immune function, and release a wide array of inflammatory mediators and cytokines.^55^ Therefore, TAMs are considered as bridges that link inflammation and tumours.^56^

It has been confirmed that glioma‐derived macrophage colony‐stimulating factor (M‐CSF) induces microglia and macrophages towards the M2 phenotype, thereby increasing the rate of tumour growth.^57^ Another study suggested that the level of M‐CSF was upregulated in both glioma tissue and its serum, and that it induced angiogenesis in vivo and in vitro through the macrophage/microglia‐secreted insulin‐like growth factor binding protein 1 (IGFBP1). Notably, investigators found that spleen tyrosine kinase (SYK) activated the PI3K/Akt pathway, further leading to the NF‐κB‐dependent upregulation of M‐CSF in glioma.^58^ Thus, the upregulation of M‐CSF induces angiogenesis through a SYK‐PI3K‐NF‐κB‐dependent mechanism. Additionally, TAMs increase the expression of IL‐8 through the NF‐κB pathway to promote angiogenesis (Figure 2). Interestingly, anti‐inflammatory drugs, including pentoxifylline, pyrrolidine dithiocarbamate and dexamethasone block, the expression of the IL‐8 induced by macrophages at least partially through the NF‐κB pathway.^59^

Human cytomegalovirus (HCMV) has been shown to be associated with glioblastoma, with more than 90% of glioblastomas (GBMs) showing HCMV infection.^60^, ^61^ Previous reports have shown that HCMV pp71 is a viral protein that boosts the progression of the cell cycle and promotes the angiogenic glioma microenvironment through the induction of the stem cell factor (SCF).^62^, ^63^ Lisa et al found that pp71, by the activation of the NF‐κB pathway, led to the upregulation of SCF and induction of pro‐inflammatory responses with the upregulation of some pro‐inflammatory cytokines (IL‐8, IL‐1B, IL‐6, LIF, PTGS2 and IL‐1A), MMPs (MMP‐3, 12, 1 and 7) and angiopoietins.^64^ The poor prognoses associated with human GBM may be attributed to the selective enhancement of pp71 levels and NF‐κB activation in pro‐inflammatory environments.

Manoj et al demonstrated that T11‐target structure (T11TS), sheep red blood cell membrane protein with immune‐enhancing and cell cycle‐regulating effects,^65^, ^66^ exerted antiangiogenic and anti‐tumour functions in an animal model and clinical glioma samples.^67^ Their latest results indicate that the pro‐inflammatory cytokine expression of TNF, IL‐8, IL‐6 and NF‐κB is enhanced in glioma‐associated ECs. T11TS treatment repressed the NF‐κB signalling pathway in glioma‐induced animal models and thus induced the downregulation of pro‐inflammatory cytokines and upregulation of anti‐inflammatory cytokines, IL‐4 and IL‐10, for glioma angiogenesis elimination^68^ (Table 1). Therefore, the expression of IL‐8 and IL‐6 in the glioma‐associated ECs induced by TNF is blocked by the NF‐κB‐mediated pathway, which has important implications for anti‐angiogenesis therapy.

**Table 1:** List of NF‐κB‐dependent therapies against glioma angiogenesis

Therapeutic targets	Targeted therapies/agents	Clinical development	Antiangiogenic pathways	Related transcription factors	References
TNF, IL‐8, IL‐6 and NF‐κB	T11‐target structure	Pre‐clinical study	Repressing the NF‐κB signalling pathway to induce downregulation of pro‐inflammatory cytokines and upregulation of anti‐inflammatory cytokines		67, 68
TNF, VEGF	Thalidomide	Pre‐clinical study	Reducing inflammatory stimulation and interfering with the transcriptional regulation of NF‐κB		^71^
ROS, IL‐6, IL‐8, GROα and MCP‐1	NG and nGO	Pre‐clinical study	Suppressing proangiogenic cytokines and inhibiting the levels of ROS depending on the p53 mutation status and NF‐κB	p53	83, 84
VEGF, HIF‐1α, MMP‐2, MMP‐9	Tumour treating field	VEGF/VEGR inhibitor: Avastin (bevacizumab), Genentech	Downregulating NF‐κB, MAPK, and PI3K/Akt signalling pathways		^110^
MMP‐3 and MMP‐9	Glycitein	Pre‐clinical study	Suppressing the transcriptional activity of NF‐κB and AP‐1 on transcription of MMP‐3 or MMP‐9	AP‐1	^111^
MMP‐9	Mangiferin	Pre‐clinical study	Inhibiting the binding of NF‐κB and AP‐1 to MMP‐9 promoter	AP‐1	^112^
VEGF‐C	Bmi‐1 inhibitor	Pre‐clinical study	Blocking the transcriptional activity of NF‐κB/VEGF‐C		^119^
Resistance to anti‐VEGF	PDGF inhibitor	Pre‐clinical study	Targeting NF‐κB/snail‐dependent PDGF	Snail	122, 123
EGFR	EGFRvIII inhibitor	Pre‐clinical study	Suppressing the transcriptional activity of NF‐κB, AP‐1, and C/EBP	AP‐1, C/EBP	125, 126
VEGF, MMP‐9	Parthenolide derivative	Australian Clinical Trials: ACTRN12616000228482	Attenuating the NF‐κB, Akt phosphorylation and VEGF and MMP‐9 expression; activating mitochondrial signalling		^128^
MMP‐9, VEGF, urokinase‐type plasminogen activator receptor and urokinase‐type plasminogen activator	Anti‐p65 intrabody construct (pFv/nu)	Pre‐clinical study	Downregulating expression of p65, and NF‐κB‐dependent genes		^131^
CA IX, HIF‐1α, hypoxia, ROS and VEGFR2	Ketogenic diet	Pre‐clinical study	Reducing the activation of NF‐κB and NF‐κB‐mediated regulators; inhibiting the levels of ROS	HIF‐1α	132, 133, 134

Abbreviations: AP, activator protein; CA IX, carbonic anhydrase IX; EBP, enhancer‐binding protein; EGFR, endothelial growth factor receptor; GROα, growth‐regulated oncogene α; HIF, hypoxia‐inducible factor; IL, interleukin; MAPK, mitogen‐activated protein kinase; MCP, monocyte chemotactic protein; MMP, matrix metalloproteinase; NF‐κB, nuclear factor‐κB; NG, graphite nanoparticles; nGO, graphene oxide nanoplatelets; PDGF, platelet‐derived growth factor; PI3K, phosphatidylinositol 3‐kinase; ROS, reactive oxygen species; TNF, tumour necrosis factor; VEGF, vascular endothelial growth factor.

As early as 1991, thalidomide was shown to be a potent TNF inhibitor that inhibited NF‐κB activation with anti‐inflammatory effects; in 1994, it was demonstrated to inhibit VEGF with antiangiogenic effects.^69^, ^70^ Investigators found that thalidomide inhibited the proliferation of ECs in vitro without affecting their viability, but did not suppress the proliferation of U251 glioma cells.^71^ NF‐κB also controlled the genes related to vascular endothelial growth factor receptor (VEGFR) expression,^72^ and the anti‐inflammatory and antiangiogenic effects of thalidomide were regulated to a certain extent by NF‐κB. Thalidomide reduced inflammatory stimulation, including the production of TNF indirectly, and interfered with the transcriptional regulation of NF‐κB in ECs directly for the simultaneous inhibition of glioma angiogenesis (Table 1). The inflammatory suppression and antiangiogenic function offered by thalidomide may be beneficial for glioma patients with severe inflammatory factors infiltration‐dependent angiogenesis, which further highlights the crucial role of inflammation in angiogenesis and regulatory role of NF‐κB.

In numerous types of tumours including gliomas, the interaction between inflammation and tumours has been recognized, and inflammation is considered the ‘seventh sign of cancer’.^73^, ^74^ Growing evidence shows that TNF is a key mediator of inflammation and tumour growth. Furthermore, the NF‐κB activated by TNF further releases pro‐inflammatory and proangiogenic factors to promote tumour vessel formation and tumour cell survival. Thus, understanding the mechanisms of NF‐κB in the interaction and mutual promotion between inflammation and angiogenesis will provide new ideas for glioma treatment.

### 2.3 NF‐κB in oxidative stress‐induced glioma angiogenesis

Interactions between inflammation and angiogenesis have been observed in the course of pathological progression.^41^ One of the characteristics of the cellular inflammatory process is a respiratory burst, which generates and accumulates a large amount of extracellular reactive oxygen species (ROS), thereby preventing the invasion of pathogens.^75^, ^76^ However, the excessive accumulation of extracellular ROS leads to an imbalance of aerobic cells and tissues, called ‘oxidative stress’, which is related to ageing and several heart and vascular diseases.^77^, ^78^ Intracellular and extracellular ROS are involved in the angiogenesis process in many pathophysiological processes.^79^, ^80^ Intracellular ROS plays a crucial role in VEGF signalling in ECs.^81^ In the tumour microenvironment, the nicotinamide adenine dinucleotide phosphate (NADPH) oxidase family, plasma membrane‐bound enzymes that generate superoxide, is a major source of ROS.^82^

**Figure 3:**
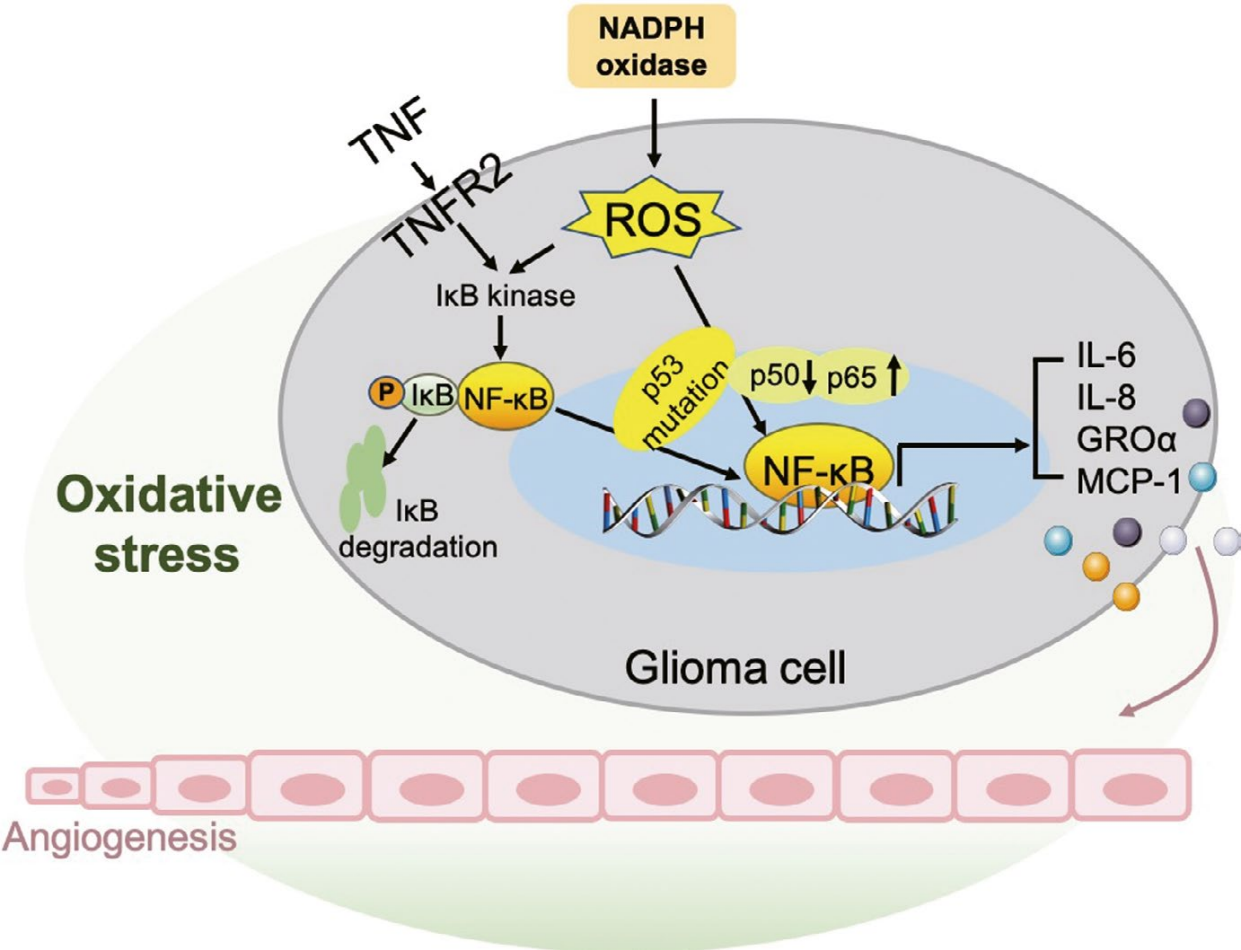
Role of NF‐κB in glioma angiogenesis under a microenvironment of oxidative stress. The ROS produced by NADPH oxidase and TNFR2 mediates the activation of NF‐κB and the main regulatory mechanism of ROS is the phosphorylation and direct oxidation of the NF‐κB subunit (reduce p50 activation and increase p65 activation), and the activation of NF‐κB further depends on the p53 mutation status. The transcriptional activity of NF‐κB for IL‐6, IL‐8, GROα and MCP‐1 promotes glioma angiogenesis. NF‐κB: nuclear factor‐κB; ROS: reactive oxygen species; IL: interleukin; TNFR: tumour necrosis factor receptor; NADPH: nicotinamide adenine dinucleotide phosphate; GRO: growth‐regulated oncogene; MCP: monocyte chemotactic protein

ROS affect the angiogenesis of tumours in numerous ways, the most important of which is the regulation of NF‐κB transcriptional activation. Since NF‐κB facilitates the synthesis of proangiogenic factors including IL‐6 and IL‐8, ROS could promote glioma angiogenesis.^46^, ^47^ The regulation of the NF‐κB signalling pathway by ROS is very complex and depends on multiple processes. However, the main regulatory mechanism is the phosphorylation and direct oxidation of the NF‐κB subunit. Since antioxidants have been shown to decrease Ser‐276 phosphorylation to inhibit p65 transcriptional activity and oxidation of p50 by ROS suppress its DNA‐binding ability, increased intracellular ROS reduce the p50 subunit activation and increase p65 subunit activation.^11^, ^83^, ^84^ The mutation status of the tumour suppressor gene p53 results in further NF‐κB activation commands.^11^, ^85^ P53‐mutated tumours tend to show a greater degree of malignancy, with enhanced invasion and reduced sensitivity to apoptotic signals.^86^ Mateusz et al stated that graphite nanoparticles and graphene oxide nanoplatelets could reduce intracellular ROS‐induced angiogenesis via the downregulation of NF‐κB‐dependent proangiogenic cytokines including IL‐6, IL‐8, growth‐regulated oncogene α (GROα) and monocyte chemotactic protein 1 (MCP‐1) in a p53wt glioma cell line (U87); however, they had no effect in a p53mut cell line (U118)^84^ (Figure 3 and Table 1).

In this regard, oxidative stress‐induced angiogenesis depends on p53 mutation status and NF‐κB regulation, which provides novel strategies in the field of nanoparticle treatment for glioma angiogenesis.

### 2.4 Caspase in NF‐κB‐dependent glioma angiogenesis

**Figure 4:**
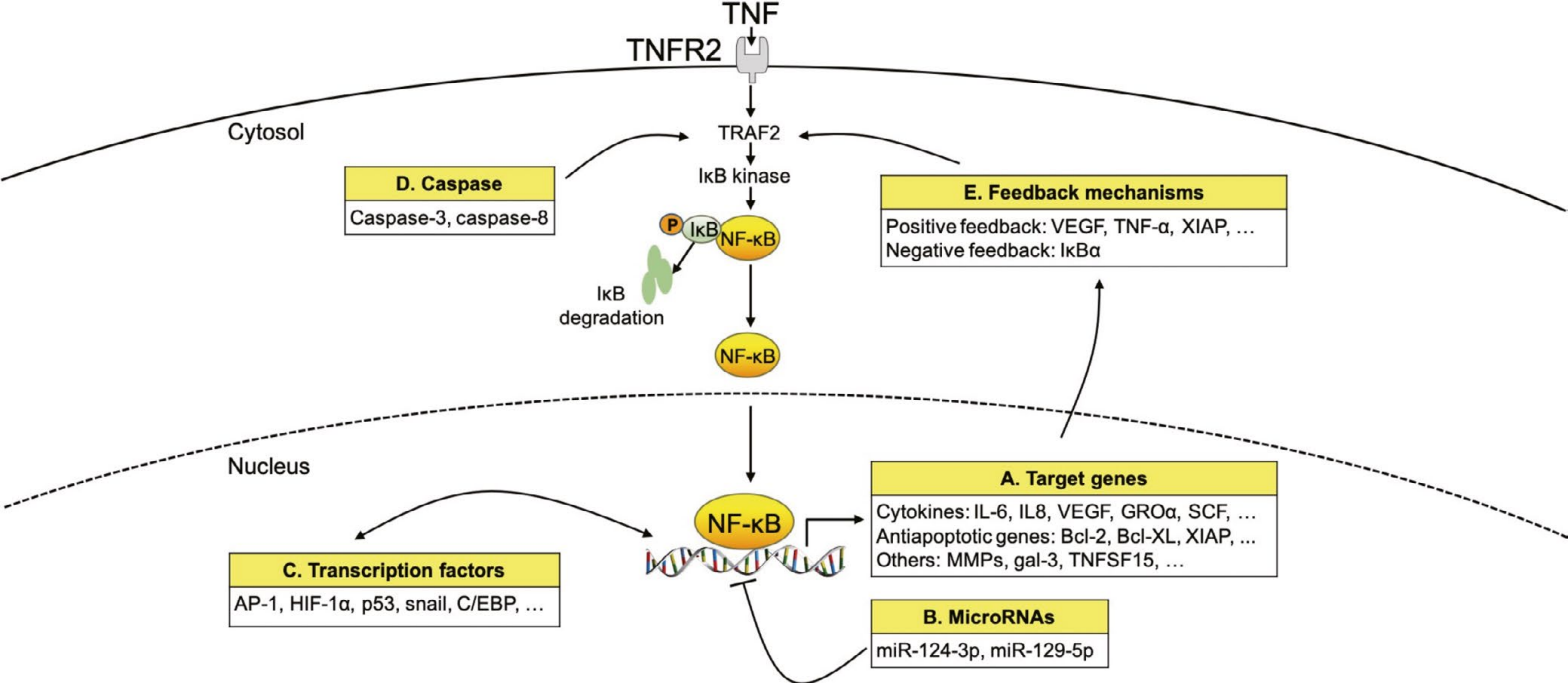
Crosstalk of the NF‐κB pathway involved in glioma angiogenesis with other signalling processes. (A) Target genes of NF‐κB associated with glioma angiogenesis include cytokines such as IL‐6, IL8, VEGF, GROα and SCF; antiapoptotic genes such as Bcl‐2, Bcl‐XL and XIAP; and other genes such as MMP, gal‐3 and TNFSF15. (B) MiR‐124‐3p and miR‐129‐5p block the NF‐κB activation pathways. (C) Numerus transcription factors such as AP‐1, HIF‐1α, p53, snail and C/EBP affect the NF‐κB activation pathways or directly activate the target genes of NF‐κB. (D) Caspase‐3 and caspase‐8 enhance the transcriptional activity of NF‐κB. (E) Positive feedback target genes of NF‐κB such as VEGF, TNF‐α and XIAP further activate the NF‐κB pathway. A significant negative feedback molecule is IκBα. GRO: growth‐regulated oncogene; SCF: stem cell factor; IL: interleukin; TNF: tumour necrosis factor; NF‐κB: nuclear factor‐κB; VEGF: vascular endothelial growth factor; XIAP: X‐chromosome‐linked inhibitor of apoptosis protein; HIF: hypoxia‐inducible factor, MMP: matrix metalloproteinase; TNFSF: tumour necrosis factor super family; Gal‐3: galectin‐3; AP: activator protein; EBP: enhancer‐binding protein

Caspase‐8 was initially identified as participating in death receptor‐induced apoptosis.^87^ Apoptosis signalling is usually absent in cancer, and caspase‐8 expression is also suppressed.^88^, ^89^ However, caspase‐8 shows high expression in glioma and may be associated with poorer prognoses. In glioma models, caspase‐8 could facilitate the expression of NF‐κB‐dependent proangiogenic cytokines and tumour promoters.^90^ Further, it has been confirmed that it exerts growth‐promoting effects in several conditions, such as fibrosis,^91^ wound healing, tissue regeneration^92^ and tumour reunion.^93^ Feng et al found that dying glioma cells, following radiation, built a proangiogenic microenvironment by the caspase 3‐dependent NF‐κB/COX‐2/PGE axis.^94^ These results demonstrate that certain cancers such as glioma may reverse caspase‐8 or the caspase‐3 pro‐apoptotic function that is dependent on NF‐κB, leading to the promotion of blood vessel formation (Figure 4).

### 2.5 MicroRNAs in NF‐κB‐dependent glioma angiogenesis

MicroRNAs (miRNAs) are a type of small, non‐coding RNA molecules that participate in cell differentiation, maturation and signal transduction by imperfectly base‐pairing with complementary sites of their target genes, leading to target mRNA degradation or translation inhibition.^95^ Zhang et al found that miR‐124‐3p was reduced in human glioma, which led to the negative regulation of neuropilin‐1 (NRP‐1). NRP‐1 is expressed as a multifunctional receptor in various human tumours, including gliomas, and the degree of expression is related to the clinicopathological characteristics of the host tumours.^96^ The expression of p‐PI3K, p‐Akt and p‐p65 (NF‐κB) was markedly reduced when miR‐124‐3p was overexpressed in glioma cells compared to the control group, and the total protein levels of PI3K, Akt and p65 were unchanged. These authors also found that the overexpression of miR‐124‐3p led to the inhibition of glioma development and blood vessel formation in vivo, in a glioma‐bearing patient‐derived xenograft (PDX) model. Therefore, the overexpression of miR‐124‐3p significantly inhibited glioma cell growth and angiogenesis by targeting the PI3K/Akt/NF‐κB pathway in both in vitro and in vivo PDX models.^97^

Similarly, other investigators suggested that the overexpression of miR‐129‐5p in glioma cells medium culturing HBMVEC showed fewer capillaries, branches and shorter tube lengths, and that the overexpression of miR‐129‐5p in glioma cells was associated with lower VEGF expression than that in the control group.^98^ MiR‐129‐5p overexpression obviously reduced the MMP‐2 and MMP‐9 protein levels and the luciferase activities of NF‐κB, indicating that miR‐129‐5p blocked the NF‐κB pathways to suppress glioma angiogenesis and growth (Figure 4).

There is an urgent need to investigate the effects of dysregulated miRNAs on NF‐κB‐induced angiogenesis in glioma, to gain a better understanding of the biological basis of the occurrence and development of glioma angiogenesis.

### 2.6 Target NF‐κB‐dependent angiogenesis for glioma therapy

#### 2.6.1 Matrix metalloproteinase

Angiogenesis is related to invasion and is used for glioma grading.^99^ A recent study showed similar molecular mechanisms for angiogenesis and invasion.^100^ The new formation of blood vessels can be considered to an invasive course in which activated ECs proliferate, adhere to the ECM molecules and migrate.^101^ MMPs are involved in angiogenesis, invasion and ECM degradation for the promotion of tumour development.^102^, ^103^, ^104^ Among MMPs, MMP‐2 and MMP‐9 have been indicated as having an upregulated expression in glioma. The upregulation and activation of MMP‐2 in association with HIF‐1α expression enhance tumour cell infiltration and blood‐brain barrier permeability.^105^ MMP‐1 and MMP‐3 levels also increase as the tumour grade increases.^106^, ^107^ It is also to be noted that the NF‐κB binding sites in the MMPs promoter regions are closely related to tumour cell invasion and angiogenesis.^108^ Therefore, effective MMP inhibitors may show promise for use in therapeutic strategies for glioma angiogenesis (Table 1).

The use of tumour treating field (TTF) therapy, entailing an alternating electric field with an intermediate‐frequency (100‐300 kHz) for tumour treatment, led to glioma suppression.^109^ It has been found that TTF suppresses the metastatic ability of glioma by the downregulation of the NF‐κB, MAPK and PI3K/Akt signalling pathways. Second, TTF application decreases the levels of VEGF, HIF1α, MMP‐2 and MMP‐9 via the suppression of NF‐κB, thus suppressing glioma angiogenesis. These results suggest that TTF is an effective MMP and NF‐κB‐related treatment for glioma invasion and angiogenesis.^110^

Glycitein, a bacterial metabolite of the isoflavone glycitein, inhibits the expression of MMP‐3 and MMP‐9 in phorbol myristate acetate (PMA)‐stimulated U87MG glioma cells. Furthermore, glycitein suppresses the transcriptional activity of NF‐κB and AP‐1 for MMP‐3 and MMP‐9.^111^ A previous study focused on mangiferin, a natural polyphenol compound isolated from *Anemarrhena asphodeloides*, which could be widely found in several higher plants including *Mangifera indica L*.^112^ Mangiferin specifically suppresses MMP‐9 mRNA and protein expression in PMA‐stimulated U87MG, U373MG and CRT‐MG glioma cells. Further mechanistic studies indicated that mangiferin inhibits MMP‐9 by the inhibition of the binding of NF‐κB and AP‐1 to MMP‐9 promoters to block glioma invasion and angiogenesis.^113^

MMPs have a vital role in the invasion and angiogenesis of malignant glioma. The inhibition of NF‐κB DNA‐binding activity and the interference of NF‐κB‐activated signal cascade leading to the inhibition of MMPs gene expression may be a promising therapeutic strategy for the blocking of glioma angiogenesis.

#### 2.6.2 Vascular endothelial growth factor

ECs are stimulated by VEGF or adhere to ECM molecules, leading to the augmentation of anti‐apoptotic genes via the PI3K/Akt or NF‐κB signalling pathways.^18^, ^19^ Therefore, anti‐angiogenesis therapy, that is, the inhibition of tumour‐associated ECs, has become a major strategy for tumour treatment.^88^ VEGF blocking and VEGF receptor inhibition have been used as anti‐angiogenesis therapies for glioma; however, their effects are weak.^114^, ^115^ B cell–specific moloney murine leukemia virus integration site 1 (Bmi‐1) is expressed in numerous cancers types, such as breast, lung and ovarian cancers, and could serve as an oncogene.^116^, ^117^, ^118^ Jiang et al found that the upregulation of Bmi‐1 induced the expression of NF‐κB target genes by the activation of NF‐κB‐induced VEGF‐C, which plays a major role in angiogenesis, thus promoting glioma angiogenesis in vitro and in vivo. It is worth noting that the angiogenesis and VEGF‐C stimulated by the upregulation of Bmi‐1 were significantly blocked after the inhibition of NF‐κB activity.^119^ VEGF‐C plays an important role in angiogenesis and EC growth and survival. It is upregulated in glioma and involved in tumour progression and prognoses^120^ (Table 1). These findings indicate that Bmi‐1 could promote angiogenesis in glioma via NF‐κB/VEGF‐C, further suggesting that NF‐κB/VEGF‐C‐dependent Bmi‐1 may represent a novel therapeutic target for antiangiogenic strategies aimed at glioma.

#### 2.6.3 Platelet‐derived growth factor (PDGF)

PDGF, a proangiogenic factor, is the major mitogen for many mesenchymal‐derived cell types, such as fibroblasts and pericytes.^121^ PDGF‐mediated endothelial‐mesenchymal transformation (EMT) reduced the expression of VEGFR‐2 in ECs. With the loss of VEGFR‐2 expression, ECs convert to a VEGF‐independent state for the maintenance of their growth and survival in glioma, leading to the resistance of ECs to anti‐VEGF treatment.^122^ The expression of snail, a pivotal downstream regulator of EMT in the glioma environment, is regulated by NF‐κB.^123^ In addition, PDGF induces NF‐κB‐dependent snail expression, resulting in resistance to anti‐VEGF treatment with the downregulation of VEGFR‐2. The inhibition of PDGF receptor sensitized VEGF/VEGFR‐2 targeted therapy in glioma‐bearing mice model (Table 1). Collectively, targeting NF‐κB/snail‐dependent PDGFs may serve as a promising strategy for cases with resistance to anti‐VEGF in glioma.

#### 2.6.4 Epidermal growth factor receptor (EGFR)

The amplification of the EGFR gene occurs in almost half of all glioblastoma cases and is related to gene rearrangement.^124^ The rearrangement is often related to activating mutations such as the loss of exons 2‐7 (EGFRvIII or EGFRde2‐7). EGFRvIII overexpression in human glioma cells or primary mouse astrocytes can lead to the significantly faster formation of tumours in animal models by intracranial or subcutaneous injection than in the control group, demonstrating that EGFRvIII enhances the carcinogenic capacity.^125^ Bonavia et al suggested that EGFRvIII facilitated high levels of IL‐8 expression in glioma clinical samples and cell lines mediated by NF‐κB, AP‐1 and C/EBP. Additionally, the knocking down of NF‐κB suppressed the EGFRvIII overexpressing glioma cell bearing‐tumour growth in vivo with the inhibition of angiogenesis, indicating the crucial role of NF‐κB in EGFRvIII enhancing the carcinogenic capacity^126^ (Table 1). In conclusion, EGFRvIII facilitates glioma angiogenesis and growth by the NF‐κB pathway. Thus, the inhibition of EGFR gene amplification and kinase activating mutants or the targeting of a unique EGFR epitope such as EGFRvIII with monoclonal antibodies for the inhibition of NF‐κB as well as tumour angiogenesis and growth may be rational strategies for the development of glioma therapy.

#### 2.6.5 Other NF‐κB ‐targeted therapeutic strategies for glioma angiogenesis

Parthenolide reportedly has the potential to cross the blood‐brain barrier and alleviate brain inflammation.^127^ Nakabayashi et al found that parthenolide inhibited U87MG glioma cell proliferation and invasion and induced angiogenesis in a dose‐dependent manner in vitro. Mechanically, parthenolide attenuates NF‐κB transcriptional activity and the expression of NF‐κB targets, VEGF and MMP‐9, in glioma cells. Moreover, parthenolide suppresses Akt phosphorylation and activated mitochondrial signalling, demonstrating that it inhibits angiogenesis by the inhibition of NF‐κB, and further suppresses glioma growth by the inhibition of the Akt signal and activation of the apoptosis signal.^128^

The transcriptional activity of NF‐κB can be induced by IκB cytoplasmic segregation and RelA/p65 phosphorylation.^129^, ^130^ Using the phage display technique to frame a single‐chain fragment of anti‐p65 antibody variable region (scFv), investigators cloned the scFv‐encoding sequence into the mammalian nuclear‐targeting vector, pCMV/myc/nuc, to fabricate an anti‐p65 intrabody construct (pFv/nu). U251 and U87 glioma cells transfected with pFv/nu dramatically suppressed the expression of p65, and NF‐κB‐dependent genes such as MMP‐9, VEGF, urokinase‐type plasminogen activator receptor and urokinase‐type plasminogen activator. Additionally, U251 and U87 glioma cells transfected with pFv/nu‐bearing intracranial tumours were almost restrained.^131^ Thus, inhibition of the transcriptional activity of NF‐κB by nuclear‐targeting intrabody could serve as a promising antiangiogenic strategy for glioma.

The ketogenic diet (KD) is a novel high‐fat, low‐carbohydrate, protein‐rich diet that targets tumour metabolism and has been used in non‐drug therapy for intractable epilepsy. Of note, first, mouse glioma models fed a KD showed higher survival values than those on a normal diet.^132^ Glioma models fed a KD at will demonstrated observable reductions in NF‐κB activation and reductions in the levels of NF‐κB‐mediated regulators in the hypoxic context, such as carbonic anhydrase IX (CA IX) and HIF‐1α.^36^ Second, the KD inhibits the levels of ROS, which boosts angiogenesis by the activation of NF‐κB transcription in tumours.^25^ Third, the KD blocks the expression of VEGFR2, the major receptor involved in tumour angiogenesis regulation^133^, ^134^ (Table 1). KD therapy that targets tumour metabolism and represses the NF‐κB‐mediated hypoxic response may provide a low‐toxic, easy‐to‐implement method for glioma aimed at angiogenesis inhibition.

## 3 CLINICAL RELEVANCE AND FUTURE PERSPECTIVES

Bevacizumab, a recombinant humanized anti‐VEGF monoclonal antibody, is the only FDA‐approved anti‐glioblastoma angiogenesis drug.^135^ Although it has been used in clinical treatment, it usually causes serious adverse reactions, and its clinical efficacy remains controversial.^136^ Bevacizumab increases the hypoxic area and boosts the rate of MMP‐2 activation, resulting in a more invasive, treatment‐resistant glioma state.^137^ Whereas the inhibition of NF‐κB transcriptional activation reduces hypoxia‐induced angiogenesis and the levels of NF‐κB‐mediated regulators in hypoxic context. In this regard, if the beneficial effects of bevacizumab can be mimicked by the inhibition of the transcriptional activity of NF‐κB in vivo, it could provide a low‐toxic method for glioma to block angiogenesis even with the inhibition of the invasive potential.

Structurally and functionally altered glioma blood vessels may impede the delivery of therapeutic agents, promote the outward leakage of tumour cells, and aid in the rapid infiltration of numerous inflammatory cells.^138^ Chronic inflammation leads to oedema development around the tumour, which aggravates the pathological progression of glioma. Although dexamethasone is used to treat glioma peripheral inflammation and oedema, its severe side effects, such as electrolyte disorders, osteoporosis, mental excitement, elevated blood pressure, menstrual disorders and weight gain, distinctly lower the quality of life and even interfere with the effectiveness of adjuvant chemoradiotherapy in glioma settings.^139^, ^140^ Based on the current literature, it is clear that the key role of NF‐κB is the interaction and mutual promotion between inflammation and angiogenesis that it offers. Thus, the inhibition of the transcriptional activity of NF‐κB may restrain the continuous recruitment and permeation of inflammatory cells, which may inhibit angiogenesis as well as peripheral inflammation and oedema development. NF‐κB is associated with angiogenetic signal transduction pathways that regulate hypoxia, oxidative stress and the production of pro‐inflammatory cytokines, proangiogenic cytokines and MMPs. Therefore, targeting NF‐κB could be a potential method for the simultaneous targeting of multiple glioma features.

Considering the wide range of the regulatory responses covered by NF‐κB, which also mediates tumour cells infiltration, proliferation and migration besides angiogenesis, the more reasonable disposal of specific subsets of NF‐κB responses could show greater efficacy in glioma treatment, in terms of angiogenesis inhibition. In addition, accumulating evidence indicates that NF‐κB participates in numerous targeted therapies, including those with MMPs, VEGF and PDGF. Further clinical study of the molecular mechanisms between angiogenesis and NF‐κB in glioma is warranted to broaden the options of targeted therapies for the prevention of NF‐κB‐dependent angiogenesis.

## 4 CONCLUSIONS

Angiogenesis in glioma accelerates tumour growth and increases the degree of malignancy. NF‐κB plays a pivotal role in the growth and progression of glioma angiogenesis. Interference with the transcriptional activity of NF‐κB that leads to alterations in the proangiogenic context and the inhibition of proangiogenic gene expression may be promising therapeutic strategies aimed at glioma angiogenesis blocking.

## CONFLICT OF INTEREST

The authors have declared no conflicts of interest.

## AUTHOR CONTRIBUTION

JJT and YLF drafted the manuscript. DFH, XWT, HFJ, XG, XMW, WMH and WW revised the manuscript.

## Data Availability

No new data generated.
